# Advances in Stigmasterol on its anti-tumor effect and mechanism of action

**DOI:** 10.3389/fonc.2022.1101289

**Published:** 2022-12-12

**Authors:** Xiaoyu Zhang, Jiayun Wang, Lin Zhu, Xuezhen Wang, Feifei Meng, Lei Xia, Hairong Zhang

**Affiliations:** ^1^School of Chinese Medicine, Shandong University of Traditional Chinese Medicine, Jinan, China; ^2^Department of Pathology, Shandong University of Traditional Chinese Medicine, Jinan, China; ^3^Department of Obstetrics and Gynecology, Shandong Provincial Third Hospital, Jinan, China

**Keywords:** stigmasterol, tumor, mechanism, pathway, plants

## Abstract

Stigmasterol is a phytosterol derived from multiple herbaceous plants such as herbs, soybean and tobacco, and it has received much attention for its various pharmacological effects including anti-inflammation, anti-diabetes, anti-oxidization, and lowering blood cholesterol. Multiple studies have revealed that stigmasterol holds promise as a potentially beneficial therapeutic agent for malignant tumors because of its significant anti-tumor bioactivity. It is reported that stigmasterol has anti-tumor effect in a variety of malignancies (e.g., breast, lung, liver and ovarian cancers) by promoting apoptosis, inhibiting proliferation, metastasis and invasion, and inducing autophagy in tumor cells. Mechanistic study shows that stigmasterol triggers apoptosis in tumor cells by regulating the PI3K/Akt signaling pathway and the generation of mitochondrial reactive oxygen species, while its anti-proliferative activity is mainly dependent on its modulatory effect on cyclin proteins and cyclin-dependent kinase (CDK). There have been multiple mechanisms underlying the anti-tumor effect of stigmasterol, which make stigmasterol promising as a new anti-tumor agent and provide insights into research on its anti-tumor role. Presently, stigmasterol has been poorly understood, and there is a paucity of systemic review on the mechanism underlying its anti-tumor effect. The current study attempts to conduct a literature review on stigmasterol for its anti-tumor effect to provide reference for researchers and clinical workers.

## 1 Introduction

Tumor, featuring a high rate of recurrence and mortality, represents one of the major threats to the health and life of human. According to the latest data released by an official journal of the American Cancer Society, there were approximately 18.1 million new cancer cases and 9.6 million cancer-related deaths globally (1). Under this background, cancer is becoming a growing public safety problem (2). Thus, much attention has been focused on looking for new effective therapeutic schemes for malignancies and exploring the underlying anti-tumor mechanisms.

**Figure 1:**
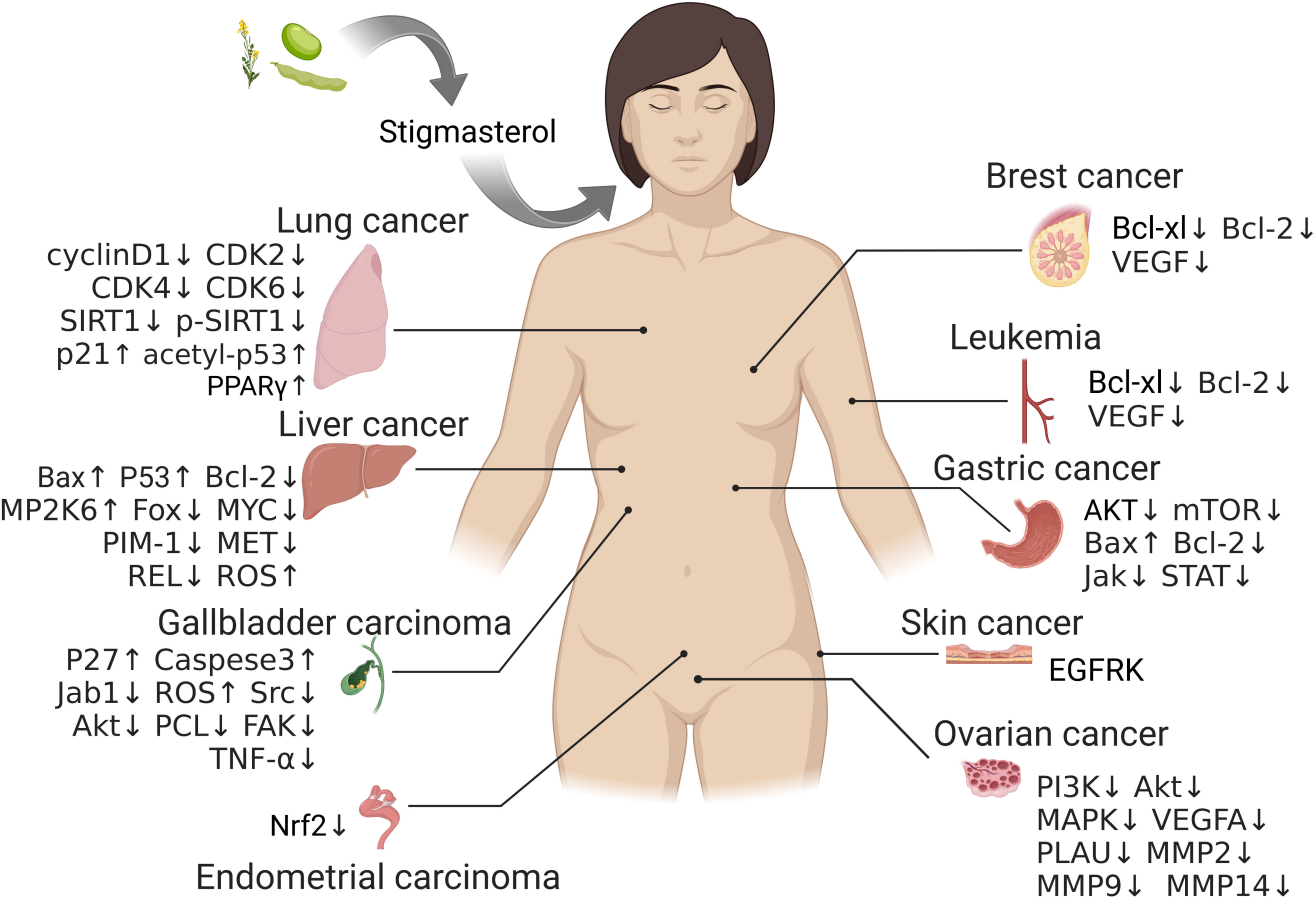
The potential targets of stigmasterol therapy in different tumors.

Phytosterol is a class of steroids containing a cyclopentanoperhydrophenanthrene skeleton, and it is widespread in plants as an important component of membranes in plant cells. It is diverse with various functions and plays a critical role in the growth and development of plants (3, 4). By now, approximately 300 types of phytosterol have been found in nature, such as campesterol, β-sitosterol and stigmasterol, which are present in most plants (5). Stigmasterol is widely distributed in multiple plants and abundant in herbs, soybean and tobacco (6, 7). It has been extensively applied in fields like medicine, foods and cosmetics owing to its high nutritional value, potent bioactivity and multiple medicinal effects, and thus it is one of the hot topics in current research on drug development from natural products. Studies have unraveled various biological and pharmaceutical properties of stigmasterol, such as analgesia (8), anti-inflammation (9–14), anti-oxidization (15, 16), anti-diabetes (15, 17, 18), maintaining psychiatric status (19), lowering blood cholesterol level (20, 21), improving learning and memory ability (22), and protecting against Leishmania (6), etc. Moreover, stigmasterol is recently reported with anti-tumor potential either *in vivo* or *in vitro* in several cancers (e.g., lung cancer (23, 24), liver cancer (25–27), gallbladder cancer (28, 29), gastric cancer (30, 31), and ovarian cancer (32)) *via* inhibiting growth while promoting apoptosis in tumor cells (**Figure 1**).

As the research on pharmacological effect of stigmasterol goes deeper, its anti-tumor activity has received much more attention in scientific researchers. With the current research results, stigmasterol has significant anti-tumor effect under multiple mechanisms and has wide clinical applications (**Table 1**). However, there is a paucity of systemic literature review. The present study reviews the mechanisms of action of stigmasterol for treatment of malignant tumors so as to provide reference for future tumor treatment.

**Table 1:** Real modules, possible mechanisms, doses and reference of Stigmasterol in various cancers.

Cancers	Real modules (animal/cell)	Possible mechanisms	Doses	Reference
Liver Cancer	HepG2	Apoptosis (Bax, p53, Bcl-2)	20μM	38
Liver Cancer	SMMC-7721, BEL-7402, H22, Kungming mice	Proliferation, Apoptosis (G0-G1, MAP2K6)	100mg/L	26
Liver Cancer	SMMC-7721	Proliferation (MAP2K6)	100mg/L	27
Liver Cancer	SMMC-7721	Apoptosis (ROS, Ca+)	64μmol/L	28
Lung Cancer	PLA-801D, A-549, H661, SK-SEM-1, BEAS-2B	Proliferation, Apoptosis (RORC)	20μg/mL	24
Lung Cancer	NCI-H1975, Nude Mouse	Proliferation (cyclinD1, CDK2, CDK4, CDK6, p21, p53, SIRT1, p-SIRT1, PPARγ)	40 mg/kg	25
Gall bladder carcinoma	Cells from the patient sample obtained from SGPGI	Apoptosis (MMP, ROS, Caspase3, p27, Jab1, G1)	17.5µM	29
Cholangiocarcinoma	KKU-M213, RMCCA-1	TNF-α, VEGFR-2	5μM,10mg/kg	30
Gastric cancer	SGC-7901, MGC-803	Proliferation, Apoptosis, Autophagy (Akt/mTOR)	10μM,20μM	31
Gastric cancer	SUN-1	Apoptosis (G2/M, Bax, Bcl-2,JAK/STAT)	15μM	32
leukemia	Jurkat, E6-1	Apoptosis (PTKs, EGFRK)	NA	82
Skin cancer	Swiss albino mice	ROS, DNA damage	200 and 400 mg/kg	86
Breast cancer	MCF-7, MCF10A, Female Balb/c mice	Apoptosis, Proliferation, (Bcl-2, Bcl-xl)	20 µM	94
Breast cancer	LMM3, Female Balb/c mice	VEGF	50 µM	99
Endometrial cancer	Ishikawa, SPEC2, MDA-MB-231,10 cases of normal endometrium,90 cases of endometrial cancer	Apoptosis (Cisplatin, Nrf2)	20μg/mL	105
Ovarian cancer	ES2, OV90	Apoptosis, migration (ROS, calcium, ER-mitochondrial axis, VEGFA, PLAU, MMP2, MMP14)	20 µg/mL	33

## 2 Stigmasterol biosynthetic pathway

**Figure 2:**
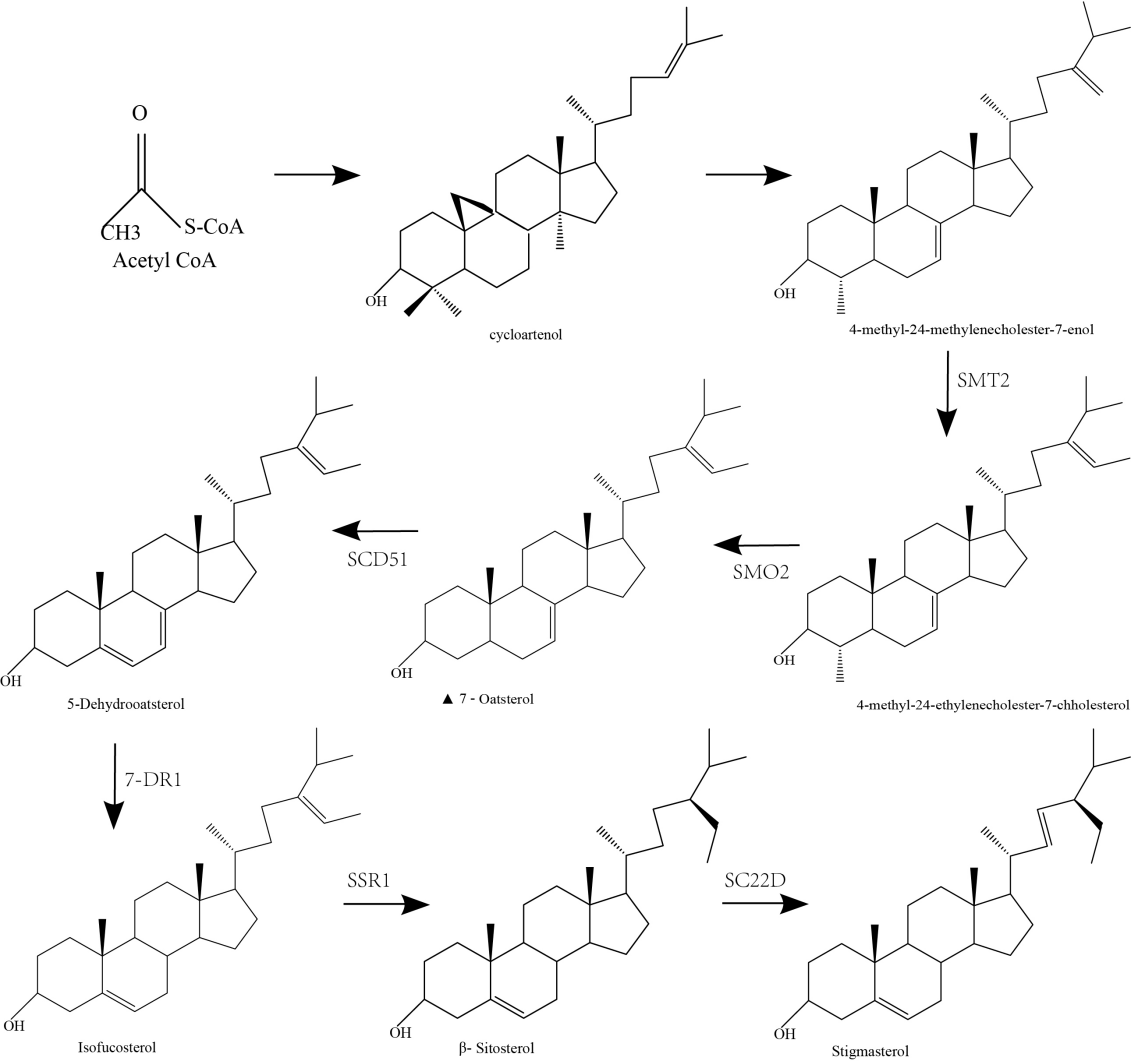
The biosynthetic pathway of stigmasterol.

Stigmasterol and β-sitosterol are basically similar in structure, whereas there is a double bond between C22 and C23 positions of the stigmasterol side chain. In most cases, acetyl-CoA is converted to cycloartenol and then to 4-methyl-24-methylene cholesteric-7-enol. The 4-methyl-24-methylene cholesteric-7-enol is subsequently converted to 4-methyl-24-ethyl-7-cholestenol *via* introduction of a second methyl group under the action of SMT2, a gene key to the synthesis of plant sterols (33). Then, SMO2 catalyzes demethylation of 4-methyl-24-ethyl-7-cholestenol at C4 position, yielding Delta-7-Avenasterol. The Delta-7-Avenasterol undergoes dehydrogenation at C5-C6 positions under the catalysis of SC5D1 to generate 5-dehydrogenated avenasterol, which is then converted to β-sitosterol following sequential reduction of the C7-C8 and C24-C28 double bonds under the action of 7-DR1 and SSR1, respectively. Eventually, the β-sitosterol is dehydrogenated to stigmasterol under the catalysis of sterol C22-desaturase (22-SD) at C22 and C23 positions (**Figure 2**).

Chemical synthesis process from acetyl-CoA to stigmasterol.

## 3 Role of stigmasterol in different cancers

### 3.1 Stigmasterol in liver cancer

Liver cancer is one of the common malignancies with a poor prognosis. The 5-year survival rate in cases with an advanced liver cancer was estimated ≤ 5%, posing a serious threat to the health and life of human (34). Additionally, it was reported that the annual incidence of liver cancer in females continued to increase by over 2% (35). Stigmasterol as one of the representative components of phytosterol is critical in liver cancer.

Apoptosis is a form of programmed cell death that occurs under both physiological and pathological conditions, and it plays a vital role in the occurrence and development of tumor (36). KIM et al. (37) found that stigmasterol up-regulated the expression of pro-apoptotic genes (Bax, p53) and down-regulated the expression of anti-apoptotic gene Bcl-2 in liver cancer cells HepG2. In the meantime, they also noted an increase in the number of apoptotic HepG2 cells in experiments including Hoechst staining, Annexin V staining and cell cycle analysis.

Proliferation as one of the basic cell functions that underlies life is a precise, ordering process under strict control (38). Tumor cells display an unrestricted proliferation, while modulating cell cycle can inhibit proliferation and induce differentiation or death in tumor cells (39). Current anti-tumor drugs act mostly *via* regulating the cell cycle process in tumor cells (40). The study of Zhang et al. (25) revealed that stigmasterol was able to induce cell arrest in G0-G1 phase (stationary phase), resulting in few cells in the G2/M phase (division phase). In addition, the authors also noted up-regulated protein expression of protein kinase MAP2K6, an important participant in cell cycle arrest. The results indicate that stigmasterol suppresses growth of liver cancer cells possibly *via* promoting cell cycle arrest. Another study (26) applied GeneChip technique to explore the target genes involved in the inhibitory effect of stigmasterol on growth of SMMC-7721 cells in human liver cancer. It was noted that stigmasterol inhibited the *in vitro* growth of SMMC-7721 cells in a time- and dose-dependent manner. Expression analysis demonstrated that stigmasterol decreased the expression of oncogenes (FOS, MYC, RAS, PIM-1, MET, REL) and increased the expression of tumor-suppressor genes (NF-2, MAP2K6) to normal levels. Combining the results, the authors held the view that stigmasterol exerted marked suppressive effects on liver cancer cells SMMC-7721 *in vitro* with the involvement of multiple target genes and intra- and extra-cellular signal transduction pathways.

Currently, there are three major apoptotic signaling pathways: mitochondrial pathway, death receptor pathway and endoplasmic reticulum pathway, among which the mitochondrial pathway is particularly important (41). Mitochondria are the main sources of ROS and the targets of pro-apoptotic actions. Ca^2+^ is an important second messenger involved in various death signal transductions, and it is intricately linked with mitochondrial function and ROS (42, 43). Li et al. (27) found that stigmasterol induced a range of apoptosis-related changes in human liver cancer cells SMMC-772, which was speculated to be achieved mostly *via* the mitochondrial pathway. Upon a stimulation, the mitochondria were damaged, which impaired the redox system and induced the production of a massive quantity of ROS, leading to a decline in mitochondrial membrane potential (ΔΨm) and extracellular Ca^2+^ influx. As a consequence, the concentration of intracellular Ca^2+^ continued to increase, triggering a series of cascade reactions and eventually apoptosis in cancer cells. The authors believed that stigmasterol had a significant suppressive effect on proliferation of SMM-7721 cells in human cancer, and it could induce apoptosis in tumor cells through promoting the oxidation by ROS, decreasing ΔΨm, increasing intracellular Ca^2+^ concentration and advancing cell cycle arrest.

### 3.2 Stigmasterol in lung cancer

Lung cancer is a malignancy originating in the bronchial mucosal epithelium and gland and featuring strong invasion, easy metastasis and recurrence (44, 45). On a global scale, lung cancer ranks second in all cancer types in terms of incidence, while it is listed first in mortality (46). According to the existing literature, drugs from natural plants have favorable therapeutic efficacy against lung cancer (47–49).

Retinoic acid-related orphan receptor C (RORC) is a DNA-binding transcription factor belonging to the family of orphan nuclear receptors (50). It has received much attention owing to its key role in regulating cell proliferation, metastasis, and chemoresistance in diverse malignant tumors (51–53). Dong et al. (23) found that stigmasterol inhibited proliferation and promoted apoptosis in lung cancer cells. The authors also noted that stigmasterol directly targeted the expression of RORC in lung cancer, and overexpression of RORC reversed the suppressive effect of stigmasterol on cancer cells. This study suggests the functional role of the stigmasterol-RORC axis in lung cancer progression, which provides a potential target for cancer treatment.

Non-small cell lung cancer (NSCLC) comprises approximately 80% of total lung cancers, while lung adenocarcinoma (LUAD) is the most common subtype of NSCLC (54). The study of Song et al. (24) performed *in vivo* and *in vitro* experiments to investigate the regulatory role of stigmasterol in LUAD and try to clarify the corresponding molecular mechanism of action. They found that stigmasterol distinctly inhibited the viability of NCI-H1975 cells but promoted lipid deposition. In the meantime, reduction of energy metabolism in cancer cells was observed, which affected the cell proliferation and colony formation. The authors also examined the expression of cyclin proteins using PPARγ inhibitor GW9662. As compared with the control group, the expression of cyclin D1, CDK2, CDK4, CDK6, SIRT1 and p-SIRT1 was significantly decreased in the high-concentration stigmasterol group, while the expression of p21, acetyl-p53 and PPARγ was significantly increased. The authors believed that stigmasterol suppressed the viability and tumorigenicity of cancer cells by targeting PPARγ.

### 3.3 Stigmasterol in gallbladder cancer

Gallbladder cancer is a collective term of primary malignant tumors in the gallbladder, including those in the cystic duct, the neck, body and base of the gallbladder (55). Its onset is insidious, and most patients are suffering from a middle-to-advanced disease at the time of diagnosis. As reported, the median survival time of gallbladder cancer was less than 6 months with a 5-year survival rate of only 5%, making gallbladder cancer a refractory disease in the world (56, 57). Stigmasterol has shown satisfactory therapeutic efficacy against gallbladder cancer, providing a new way in clinical treatment.

Pandey et al. (28) sampled gallbladder cancer tissue in clinical patients and found that induction of apoptosis in cancer cells was linked with Caspase-3 increase, ROS production, ΔΨm disruption, and expression of p27 and Jab1 proteins. The dose-dependent activation of Caspase-3 suggests that stigmasterol can induce apoptosis in cancer cells *via* mitochondria-mediated pathway, while the disruption of ΔΨm *via* depolarization under the action of stigmasterol in a dose-dependent fashion is considered as an essential prerequisite of activation of apoptosis (58, 59). The authors also observed that Caspase-3 inhibitor Z-DEVDFMK distinctly reduced the stigmasterol-induced cytotoxicity in cancer cells but failed to completely weaken the viability of cells. Therefore, stigmasterol might induce apoptosis in gallbladder cancer cells *via* Caspase-dependent and independent pathways. Moreover, this study also reported significant G1 arrest in cancer cells treated with stigmasterol. The study of Kangsamaksin et al. (29) revealed that stigmasterol inhibited the viability, migration and morphogenesis of human umbilical vein endothelial cells (HUVECs), whereas it had no suppressive effect on cholangiocarcinoma (CCA) cells KKU-M213. Expression experiments demonstrated that stigmasterol greatly reduced the transcriptional level of TNF-α and the protein levels of a series of downstream effectors of VEGFR-2 signaling (including Src, p-Src, Akt, p-Akt, PCL, p-PCL, FAK and p-FAK), while management of TNF-α rescued the expression of these effectors. *In vivo* experiment revealed that stigmasterol disrupted tumor angiogenesis and decreased the growth of CCA tumor graft. In addition, immunohistochemical analysis showed reductions in CD31-positive vessels and recruited macrophages after stigmasterol administration. Collectively, stigmasterol could effectively target tumor endothelial cells to inhibit CCA tumor growth with its anti-inflammatory activity, and it could be an ideal candidate agent for CCA treatment.

### 3.4 Stigmasterol in gastric cancer

Gastric cancer is a life-threatening malignancy, with its incidence ranking sixth and mortality ranking third in total malignancies globally (1). Prior investigations showed that the incidence of gastric cancer increased with age, which makes early prevention and treatment of alimentary malignancies particularly important (60). As the most common, highly heterogeneous malignancy (61), gastric cancer currently is treated by combination therapies involving surgery and adjuvant therapies such as chemotherapy and radiotherapy (62). Plant extracts have certain strengths to preventing premalignancy, prolonging survival time, relieving adverse reactions to chemotherapy, and other aspects in patients with gastric cancer. Thus, they are vital in prevention and treatment of gastric cancer (63, 64).

Autophagy is a ubiquitous, highly conserved catabolic process complementary to apoptosis, and it plays a key part in multiple biological processes such as cell development, innate immunity, stress response, and cell death (65). Zhao et al. (30) explored the role and molecular mechanism of stigmasterol in inducing autophagy in gastric cancer cells. They found that stigmasterol suppressed the proliferation of SGC-7901 and MGC-803 cells probably *via* inhibiting the Akt/mTOR signaling pathway and inducing apoptosis and autophagy. This is consistent with previous studies (66, 67). In addition, the *in vivo* experiment also proved the suppressive effect of stigmasterol on growth of xenograft tumor. Combining these results, the authors believed that stigmasterol induced apoptosis and protective autophagy in gastric cancer cells while inhibiting the Akt/mTOR signaling pathway, and they thought stigmasterol was likely to become a potential anticancer agent in future gastric cancer treatment. The study of Li et al. (31) investigated the anti-cancer effect of stigmasterol in gastric cancer and noted increased apoptosis and G2/M arrest in cancer cells SNU-1. When apoptotic cells are cleaned up from the body, cell cycle arrest impedes cell division and then induces apoptosis (68). Previous studies demonstrated that phytosterol could induce apoptosis and cell cycle arrest in tumor cells (69, 70). Another study indicated an increase in Bax protein expression while a decrease in Bcl-2 protein expression, which further proved the promoting effect of stigmasterol on apoptosis of tumor cells. Metastatic cancer is generally difficult to treat, and agents capable of preventing metastasis are considered as important for cancer treatment (71). Li et al. noted that stigmasterol was capable of inhibiting the metastatic potential of gastric cancer cells. The JAK/STAT signaling pathway is highly activated in cancer cells, with significant implications in tumor development (72). In the study of Li et al., stigmasterol was found with an inhibitory effect on the JAK/STAT signaling pathway in gastric cancer, suggesting its potential as a candidate agent for gastric cancer treatment.

### 3.5 Stigmasterol in leukemia

Leukemia is a malignancy arising from hematopoietic tissue, usually driven by aberrant proliferation of leukocytes within the bone marrow (73). Presently, therapeutic approaches for leukemia mainly include bone marrow transplantation (BMT) (74), chemotherapy (75), and immunotherapy (76). However, the current chemotherapy commonly leads to severe side effects, and patients usually respond to the therapy poorly (77, 78). In the meantime, the drug resistance of leukemia cells also limits the efficacy of multiple chemotherapeutic agents, reducing the cure rate and thereby leading to a poor outcome in patients (79). Therefore, it is particularly important to develop new treatment strategies for leukemia that can reduce side effects, prolong the survival time and improve the quality of life of patients.

Raczyk et al. (80) examined the cytotoxic effect of three stigmasteryl esters on leukemia cells using MTT assay, and they found that the stigmasteryl linoleate had the greatest cytotoxic effect. Nazemi et al. (81) explored the anti-tumor and pharmaceutical activities of stigmasterol in oral epithelial carcinoma cell line KB/C152 and T lymphoblastic leukemia cell line Jurkat/E6-1. With the PASS software, the authors confirmed that stigmasterol induced apoptosis in cells. In addition, they also found stable binding between stigmasterol and the active sites of PTKs and epidermal growth factor receptor (EGFR). Moreover, the authors also proved the good pharmacokinetic properties of stigmasterol, providing evidence for use of stigmasterol in clinical treatment of oral epithelial carcinoma and leukemia.

### 3.6 Stigmasterol in skin cancer

Skin cancer is a significant health problem increasingly prevalent in human (82, 83), and it can arise from the epidermis as malignant melanoma (MM) or non-melanoma skin cancer (NMSC). The pathogenesis of skin cancer is complex, and one known significant cause is the DNA defects resulting from UV exposure, which involves multiple mutated genes and molecular signaling pathways. Skin cancer can be found in various ethnic groups and make effects across the lifespan (84). In this context, there is an urgent need to look for plant extracts that can be employed as agents for skin cancer treatment.

Ali et al. (85) studied the chemo-preventive benefits of stigmasterol in 7,12-dimethylbenz[a]-anthracene (DMBA) -induced skin cancer in Swiss albino mice and found that stigmasterol led to tumor shrinkage and reduced the number of cumulative papillomas. Additionally, stigmasterol was found to significantly decrease the activity of serum enzymes, such as aspartate aminotransferase (AST), alanine aminotransferase (ALT), alkaline phosphatase (AP) and bilirubin, but distinctly increase the activity of glutathione, superoxide dismutase (SOD) and catalase. It could be inferred that stigmasterol has chemo-preventive property in skin cancers, and such property might be linked with oxidative stress.

Cutaneous melanoma, featuring high invasion, high degree of worsening and poor prognosis, ranks third in all skin malignancies and accounts for approximately 10% of all skin cancers (86, 87). Currently, the preferred treatment for melanoma remains surgery, which helps patients survive longer (88). Nevertheless, the incidence and mortality of melanoma are still high in spite of considerable progress in terms of therapies (89), which prompts us to look for new therapies. The study of Cheng et al. (90) revealed that stigmasterol inhibited proliferation and promoted apoptosis in melanoma cells B16-F10. After 48-72 h of stigmasterol treatment, numerous apoptosomes, decreased number of adherent cells while increased number of floating and dead cells were observed, presenting as typical presentations of apoptosis. Additionally, DAPI staining assay found a series of apoptosis-related events, such as chromatin condensation, expansion of nuclei or formation of apoptosomes in a large number of cells, after 72 h of treatment with stigmasterol. Considering all the findings in this study, stigmasterol inhibited growth of melanoma cells B16-F10 *via* inducing apoptosis to some extent.

### 3.7 Stigmasterol in breast cancer

Breast cancer is common in females and ranks first in female malignancies in terms of incidence. Despite that, the incidence of breast cancer continues to increase annually, severely affecting the quality of life of patients and inflicting a heavy burden on the patient family and society (91, 92). It is of great significance to seek for candidates with good targeting ability towards breast cancer cells and characteristics of low toxicity, high efficiency and safety. Presently, natural products are increasingly used to develop efficient breast cancer-targeting agents for clinical treatment.

AmeliMojarad et al. (93) assessed the anti-tumor effect of stigmasterol in breast cancer cell line MCF-7 and found significant reductions in the expression of anti-apoptotic genes Bcl-xL and Bcl-2. Moreover, the *in vivo* experiment in BALB/c mice revealed a significantly reduced tumor volume in mice treated with stigmasterol for 30 days in comparison to the control group, suggesting the potential therapeutic efficacy of stigmasterol for tumor. Tumor angiogenesis is definitively significant in tumor growth. Through new vessels, tumor accesses nutrients from the host and then delivers tumor cells to the host to potentiate tumor distant metastasis (94, 95). At present, anti-angiogenic therapies are undergoing clinical translation (96, 97). Michelini et al. (98) found that stigmasterol derivatives inhibited the formation of capillary-like structures and the migration in HUVECs and decreased the expression of vascular endothelial growth factor (VEGF) in IL-6-stimulated macrophages and breast cancer cells LMM3.

### 3.8 Stigmasterol in endometrial cancer

Statistically, the incidence of endometrial cancer increased at the rate of 0.69% per year from 1990 to 2019 on a global scale, and patients with endometrial cancer became younger (99). Early diagnosis is conducive to increasing the cure rate of patients, whereas there are 21% patients who are suffering from metastasis to regional lymph nodes while 9% with distant metastasis at initial diagnosis (100). For patients who are unfit for surgery or decline it, hormone therapy, chemotherapy, and targeted therapy remain the basis in clinical treatment for endometrial cancer (101). Nonetheless, the current drug therapies still present many problems, such as resistance, toxicity, and poor efficacy. Therefore, it is urgent to develop agents that are safer and more effective in improving the survival and quality of life of patients with endometrial cancer.

In recent years, increasing evidence has suggested that Nrf2 is essential in promoting tumor recurrence by increasing patient tolerance to adjuvant chemotherapy or radiotherapy (102, 103). Liao et al. (104) applied network pharmacology to find that stigmasterol might be an inhibitor of Nrf2. In addition, experimental result revealed that stigmasterol inhibited the expression of Nrf2 protein in human endometrial cancer in a dose-dependent fashion. Cisplatin acts to inhibit cell division and increase apoptosis in tumor cells by inducing unwinding and separation of double-stranded DNA (105). In addition, it also induces the mitochondrial ROS to accumulate, activating the mitochondria-dependent apoptotic pathways and then leading to apoptosis (106). However, its clinical application is constrained due to its significant ototoxicity, nephrotoxicity, and drug resistance (107). In this context, Cisplatin is usually used in combination with other agents to help reduce resistance or adverse events and then improve clinical efficacy. In the study of Liao et al., the combination of Cisplatin with stigmasterol significantly inhibited the activity of Nrf2-ARE. In addition, stigmasterol enhanced the effect of Cisplatin to inhibit cell growth, migration, and invasion, and to promote early apoptosis in endometrial cancer cells. The results indicated that Nrf2 was significant in chemoresistance in endometrial cancer, and it had potential to inhibit Cisplatin resistance as a novel potential inhibitor of Nrf2.

### 3.9 Stigmasterol in ovarian cancer

Ovarian cancer represents one of the top three malignancies of the female reproductive system with the highest rate of lethality (108). The early symptoms of ovarian cancer is atypical, and there is a paucity of effective screening methods. Besides, the ovarian is in deep pelvic cavity. All above makes most patients being suffering from a middle-to-advanced cancer at the time of diagnosis. It was reported that the 5-year survival rate associated with an advanced disease was only 29% (109, 110). Looking for safe and effective therapeutic strategies for ovarian cancer, therefore, has become a difficult but a hot topic in relevant research.

Bae et al. (32) confirmed the complicated anti-cancer effects of stigmasterol in ovarian cancer. Endoplasmic reticulum (ER) is an organelle vital in protein translocation, folding and post-transcriptional modification in eukaryotic cells. The accumulation of ER stress can induce death in tumor cells (111). It was reported that stigmasterol could activate ER sensor proteins and ER-mitochondria axis proteins in ovarian cancer cells, demonstrating that stigmasterol exerts its anti-tumor effect by regulating the ER-mitochondria axis. Additionally, stigmasterol was also reported with suppressive effect on cell cycle progress in ovarian cancer cells ES2 and OV90 *via* inhibiting their proliferation. PI3K/MAPK signaling cascade plays a key role in proliferation and cell cycle process in cancer cells (112). It is frequently activated in ovarian cancer, and thus its suppression emerges as a viable option for cancer treatment. Since anti-cancer drugs are developed targeting the malignant properties of cancer cells (113), tumor sphere models are conducive to exploring the therapeutic efficacy of these drugs. Stigmasterol can effectively inhibit the accumulation of ovarian cancer cells, while cancer cells that fail to assemble into a tumor mass display a scattered distribution. VEGFA can stimulate the mitosis and migration in ovarian cancer cells (114). PLAU can induce the migration and metastasis of breast cancer cells (115). Matrix metalloproteinases (MMPs) exhibit overexpression in multiple tumor settings to promote tumor metastasis and migration. Studies found that stigmasterol could reduce the expression levels of VEGFA, PLAU, MMP2, MMP9 and MMP14 in ES2 and OV90 cells.

## 4 Discussion

In recent years, the incidence and mortality of cancer are increasing annually, which is valued by scientific workers. According to the World Health Organization (WHO) statistics, the number of new cancer cases worldwide is expected to exceed 27 million by 2040 (91). Surgery, chemotherapy and radiotherapy are the mainstay of treatment for cancer, but there may have some side effects such as nausea, hair loss and cardiotoxicity. Besides, the treatment cost is high, and the suppressive effect towards tumor metastasis is constrained (116). Plant extracts and metabolites are considered as safer alternatives to synthetic drugs. Traditional medicine has successively applied plant extracts to treat or cure many diseases and believes that the combination of conventional treatment with plant extracts is a promising and effective therapeutic approach in cancer treatment.

**Figure 3:**
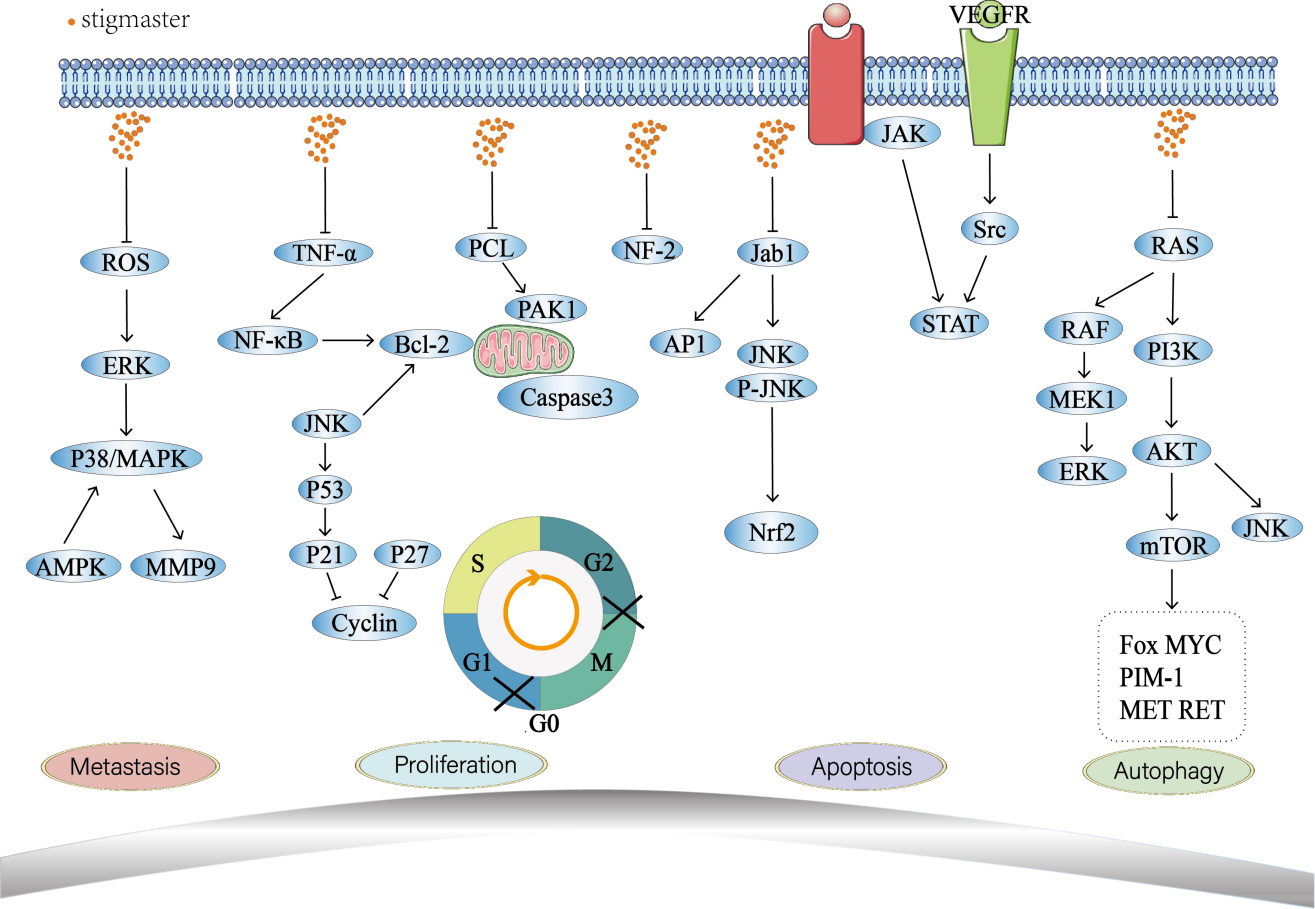
Antitumor mechanisms of stigmasterol.

Phytosterol is generally found in plant foods (e.g., vegetable oil, nut, plant seeds, vegetables, and fruits) as free sterol, phytostanyl ester, steryl glycoside (SG) or acylated SG (117). People can take phytosterol from daily diet and more from plant foods. Stigmasterol is a common phytosterol that is safe and free from oral toxicity (118). It has anti-tumor activities by regulating multiple biological behaviors of tumor cells such as apoptosis, proliferation, metastasis, invasion, and autophagy (**Figure 3**). Numerous studies have proved that inflammation is closely linked with the onset of some tumors. For example, close relationships have been confirmed between the chronic infections that are caused by viruses, bacteria or mycoplasmata and the occurrence of some tumors, such as HPV and cervical cancer (119), HBV and liver cancer (120), Helicobacter pylori and gastric cancer (121). Cytokines such as TNF-α (122), IL-1 (123) and IL-6 (124) have significant pro-inflammatory implications. Inflammatory mediators are important participants in the occurrence and development of tumor with stimulating effects on cell growth, angiogenesis, lymphangiogenesis, tumor invasion and metastasis (125). At present, the anti-inflammatory property of stigmasterol has been increasingly investigated (9–14), providing a new direction for research on anti-tumor effect of stigmasterol.

Stigmasterol combination therapy has also attracted much attention from researchers. Compared with traditional drugs, drugs based on nanomaterials have incomparable advantages of free chemotherapeutic drugs, such as good biocompatibility, reduce the toxic effect on cells, target to the tumor microenvironment, achieve sustained release of drugs and prolonged blood circulation time (126, 127). Torres et al. (128) used solid lipid nanoparticles coated with stigmasterol and found that it had good performance in the treatment of lung cancer. Stigmasterol has also shown great potential in immunotherapy (129). A study has found that stigmasterol combined with β-sitosterol can inhibit the stimulatory effect of the known stimulator lymphocyte mitogen-induced stimulatory effect, resulting in the activation of immune cells and the reduction of cytokine secretion, thus playing an immunomodulatory role (130). Stigmasterol combined with chemotherapy is also one of the directions worth studying. Gautam et al. (131) have shown that polyethylene glycol nanohybrid plant liposomes combined with chemotherapy have shown good effects in the treatment of breast cancer.

Although stigmasterol has been extensively studied for its anti-tumor mechanisms, current studies are still premature. It remains elusive about the specific targets and signaling pathways involved in the anti-tumor effect of stigmasterol, and the underlying molecular mechanism is speculated as an interplay between multiple signaling pathways. The current mechanistic studies mostly focus on one or more targets of stigmasterol, whereas systemic study is missing. Therefore, in-depth research from multiple aspects and levels is required to promote the application of stigmasterol in the field of tumor treatment. Moreover, most of the current findings are derived from *in vitro* or *in vivo* animal experiments but have rarely been clinically translated, requiring clinical trials to explore the practical applications of stigmasterol in human bodies. In the future, more targets and signaling pathways with implications in the anti-tumor effect of stigmasterol are expected to be identified.

## 5 Future perspectives

Diet has been identified as an important and modifiable risk factor for cancer. Therefore, dietary modification, including the inclusion of functional food ingredients with chemopreventive properties, has been identified as a potential strategy to stop or reverse the early stages of malignancy before its manifestation. Research have proved that functional dietary components can be used effectively for the treatment, especially for the prevention of diseases. In terms of anticancer therapy, dietary phytochemicals have attracted increasing attention due to their high efficiency and low toxicity in regulating key intracellular signaling pathways. Stigmasterol are a class of bioactive dietary phytochemicals. Studies have found that stigmasterol can promote tumor cell apoptosis, inhibit tumor cell proliferation, metastasis and invasion, and induce autophagy in a variety of malignant tumors such as breast cancer, lung cancer, liver cancer and ovarian cancer. However, the research on stigmasterol is still not in-depth.

In the future, we still have many problems about stigmasterol to explore. Firstly, researchers should substitute *in vivo* and *in vitro* experiments into clinical trials to fully explore the potential of stigmasterol in tumor treatment. Secondly, Whether derivatives or analogues of stigmasterol also play a similar role in cancer. Third, stigmasterol is poorly soluble in water, and there are few studies on novel formulations of stigmasterol. Fourth, the optimal dose of stigmasterol in the treatment of tumors needs to be studied. Fifth, whether stigmasterol, as a potent anticancer agent, will promote the therapeutic effect when combined with other anticancer methods still remains to be seen.

Stigmasterol exerts anti-tumor effects by promoting tumor cell apoptosis, inhibiting proliferation and metastasis, and inducing autophagy in tumor cells.

## Author contributions

XZ wrote the manuscript and drew the pictures. JW collected and organize literature. LZ, XW, FM proofread the manuscript. HZ and LX are fully responsible for the study designing, research fields, drafting, and finalizing the paper. All authors contributed to the article and approved the submitted version.

## Glossary

**Table:**

PI3K	phosphatidylinositol-3-kinase
AKT	protein kinase B
CDK	cyclin-dependent kinase
Bax	Bcl-2-Associated X
Bcl-2	B-cell lymphoma-2
MAP2K6	mitogen-activated protein kinase 6
FOS	Fos proto-oncogene (AP-1 transcription factor subunit)
MYC	MYC proto-oncogene
BHLH	Transcription Factor
RAS	rat sarcoma
PIM-1	Pim-1 proto-oncogene, serine/threonine kinase
MET	mesenchymal-epithelial transition factor
REL	REL proto-oncogene (NF-kB subunit)
NF-2	neurofibromatosis type 2
ROS	reactive oxygen species
RORC	retinoic acid-related orphan receptor C
NSCLC	non-small cell lung cancer
LUAD	lung adenocarcinoma
PPARγ	peroxisome proliferator activated receptor gamma
ΔΨm	mitochondrial membrane potential
CDK2	cyclin-dependent kinase 2
CDK4	cyclin-dependent kinase 4
CDK6	cyclin-dependent kinase 6
SIRT1	Sirtuin 1
Z-DEVDFMK	predominantly Caspase-3 inhibitor
HUVECs	human umbilical vein endothelial cells
CCA	cholangiocarcinoma
Src	sarcoma gene
FAK	focal adhesion kinase
TNF-α	tumor necrosis factor alpha
mTOR	mechanistic target of rapamycin
JAK	the Janus kinases
STAT	signal transducer and activator of transcription
BMT	bone marrow transplantation
EGFR	epidermal growth factor receptor
MM	malignant melanoma
NMSC	non-melanoma skin cancer
DMBA	dimethylbenz[a]-anthracene
AST	aspartate aminotransferase
ALT	alanine aminotransferase
AP	alkaline phosphatase
SODs	superoxide dismutases
Bcl-xL	B-cell lymphoma-extra-large
VEGF	vascular endothelial growth factor
Nrf2	nuclear factor erythroid 2-related factor 2
ER	endoplasmic reticulum
PLAU	plasminogen activator urokinase
MMPs	matrix metalloproteinase
VEGFA	vascular endothelial growth factor A
SG	steryl glycoside
MMP2	matrix metalloproteinase 2
MMP9	matrix metalloproteinase 9
MMP14	matrix metalloproteinase 14
HPV	human papilloma virus
HBV	hepatitis B virus
IL-1	interleukin 1
IL-6	interleukin 6
SMT2	sterol-C24-methyltransferase 2
SMO2	Sterol-4 α, Methyl oxidase
SC5D1	sterol C5 desaturase 1
7-DR1	sterol Δ, 7-Reductase1
SSR1	sterol side chain reductase1
22-SD	sterol C22-desaturase
